# A New Antimalarial Noreudesmane Sesquiterpenoid from *Dobinea delavayi*

**DOI:** 10.1007/s13659-020-00234-4

**Published:** 2020-02-24

**Authors:** Xiu-Rong Wu, Yi Shen, Shu-Jun Cui, Xiao-Lei Luo, Chao-Jiang Xiao, Bei Jiang

**Affiliations:** 1 Institute of Materia Medica, Dali University, Xueren Road 2, Dali, 671000 People’s Republic of China; 2 College of Pharmacy and Chemistry, Dali University, Xueren Road 2, Dali, 671000 People’s Republic of China

**Keywords:** *Dobinea delavayi*, Anacardiaceae, Noreudesmane sesquiterpenoid, Antimalarial activity

## Abstract

**Abstract:**

One previously undescribed angeloylated noreudesmane sesquiterpenoid, dobinin O (**1**), along with four known eudesmane sesquiterpenoids (**2**–**5**) were isolated from the peeled roots of *Dobinea delavayi*. Their structures were elucidated by extensive spectroscopic data analyses. In addition, compound **1** exhibited moderate antimalarial activity against *Plasmodium yoelii* BY265RFP with the inhibition ratio of 17.8 ± 13.3% at the dose of 30 mg/kg/day.

**Graphic Abstract:**

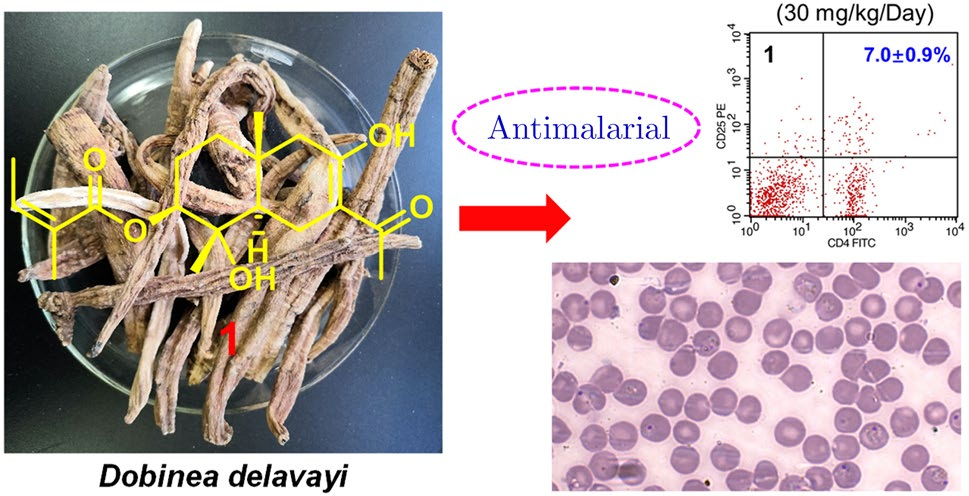

**Electronic supplementary material:**

The online version of this article (10.1007/s13659-020-00234-4) contains supplementary material, which is available to authorized users.

## Introduction

*Dobinea delavayi* (Baill.) Baill. is a perennial herb with purple-brown, cylindrical and bulky roots, which is distributed in Yunnan and Sichuan provinces of China at the altitude from 1100 to 2300 m [1]. The shape of processed roots of *D. delavyi* likes a sheep’s horn. Therefore, *D. delavyi* is called “Yang-Jiao-Tian-Ma” in China, and used to treat cough due to heat in the lung, traumatic injury, mumps, mastitis, sores, furuncles, and so on, in the folk [2].

In our previous study, *D. delavayi* showed significant antimalarial activity against *Plasmodium yoelii* BY265RFP. Subsequently, five angeloylated eudesmane sesquiterpenoid dimers dodelates A–E, with moderate antimalarial activities, were isolated from the roots of *D. delavayi* [3]. In our ongoing study, one previously undescribed angeloylated noreudesmane sesquiterpenoid, dobinin O (**1**), along with four known eudesmane sesquiterpenoids (**2**–**5**) were isolated from the peeled roots of *Dobinea delavayi*. Their structures were elucidated by extensive spectroscopic data analyses. In addition, compound **1** exhibited moderate antimalarial activity against *Plasmodium yoelii* BY265RFP with the inhibition ratio of 17.8 ± 13.3% at the dose of 30 mg/kg/day.

## Results and Discussion

Compound **1** was obtained as white powder, and assigned the molecular formula C_19_H_28_O_5_ (six degrees of unsaturation) from its HRESIMS and ^1^H and ^13^C NMR spectra (including DEPT). The ^1^H NMR data of **1** (Table 1; Fig. S1 in Supporting Information) showed one enolic hydroxyl at *δ*_H_ 15.96 (s), five methyls at *δ*_H_ 0.96, 1.33, 1.89, 1.98 and 2.17, one olefinic proton at *δ*_H_ 6.09 (1H, qq, *J* = 7.2, 1.5 Hz), and two protons from oxygenated methines or hydroxyls at *δ*_H_ 4.76 (1H, dd, *J* = 11.6, 4.3 Hz) and 3.80 (1H, s). Another nine proton resonances from either methines or methylenes were found to occur in a relatively high-field region (between *δ*_H_ 1.47 and 2.74). Analyses of the ^13^C NMR spectrum of **1** with the aid of the DEPT-90 and -135 spectra revealed the existence of 19 carbon resonances (Table 1; Fig. S2 in Supporting Information), including five methyls (*δ*_C_ 15.9, 18.0, 19.2, 20.8 and 25.3), four sp^3^ methylenes, two sp^3^ methines (including one oxy-methine at *δ*_C_ 81.6), one sp^2^ methine (*δ*_C_ 137.9), two sp^3^ quaternary carbons (*δ*_C_ 34.0 and 73.8), and five sp^2^ quaternary carbons (*δ*_C_ 106.5, 129.3, 167.7, 180.4 and 200.4). Among them, an angeloyl was recognized easily by comparing its chemical shifts with those of reported compounds [4].

**Table 1:** NMR data of compound **1** (*δ* in ppm, *J* in Hz)

Position	*δ*_H_^a^	*δ*_C_^b^
1	1.65 (overlap); 1.48 (td, 12.8, 3.6)	38.7
2	1.80 (m); 1.62 (overlap)	26.1
3	4.76 (dd, 11.6, 4.3)	81.6
4		73.8
5	1.72 (dd, 12.4, 4.6)	50.0
6	2.72 (dd, 15.3, 4.6); 2.35 (overlap)	21.6
7		106.5
8		180.4
9	2.32 (overlap); 2.06 (overlap)	50.0
10		34.0
11		200.4
12	2.17 (3H, s)	25.3
14	0.96 (3H, s)	19.2
15	1.33 (3H, s)	18.0
1′		167.7
2′		129.3
3′	6.09 (qq, 7.2, 1.5)	137.9
4′	1.98 (3H, dq, 7.2, 1.5)	15.9
5′	1.89 (3H, qui, 1.5)	20.8
4-OH	3.80 (s)	
8-OH	15.96 (s)	

^a^Measured in CD_3_COCD_3_ at 400 MHz

^b^Measured in CD_3_COCD_3_ at 100 MHz

**Fig. 1:**
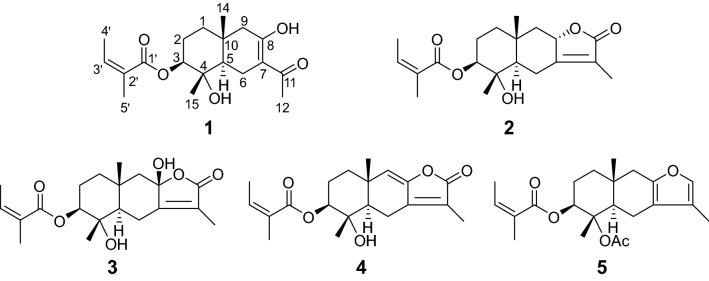
Structures of compounds **1**–**5**

**Scheme 1:**
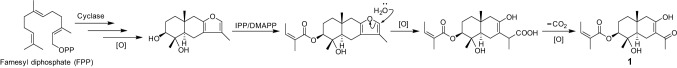
Plausible biosynthetic pathway of **1**

**Fig. 2:**
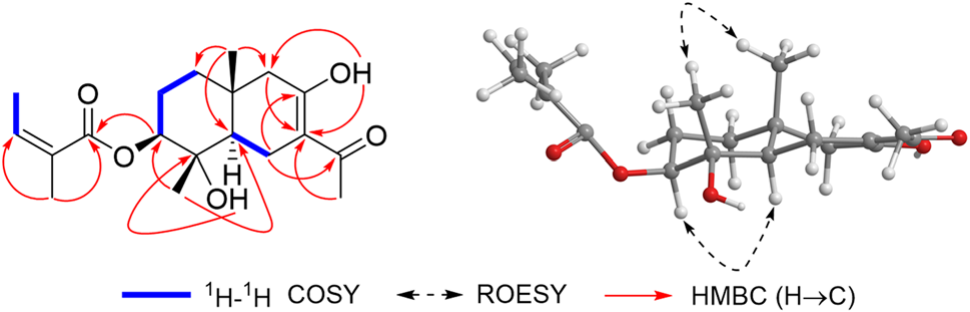
Key 2D NMR correlations of **1**

Analysis of ^1^H–^1^H COSY spectrum of **1** (Fig. 2) gave three partial structures: H_2_-1/H_2_-2/H-3, H-5/H_2_-6, and H-3′/H_3_-4′. The Me-14 (*δ*_H_ 0.96, s) showed HMBC correlations (Fig. 2) to C-1 (*δ*_C_ 38.7), C-5 (*δ*_C_ 50.0) and C-9 (*δ*_C_ 50.0), which suggested Me-14 bonded to C-10. Similarly, HMBC correlations from Me-15 (*δ*_H_ 1.33, s) to C-3 (*δ*_C_ 81.6) and C-5 suggested Me-15 jointed to C-4. HMBC correlations from the enolic hydroxyl (*δ*_H_ 15.96) to C-7 (*δ*_C_ 106.5) and C-9 suggested it jointed to C-8. Associate with HMBC correlations of H_2_-6 (*δ*_H_ 2.72, dd, *J* = 15.3, 4.6 Hz and *δ*_H_ 2.35, overlap) with C-8 (*δ*_C_ 180.4) and C-11 (*δ*_C_ 200.4), H_2_-9 (*δ*_H_ 2.32 and 2.06, both overlap) to C-7, and Me-12 (*δ*_H_ 2.17) only to C-7 and C-11, a noreudesmane sesquiterpenoid moiety in **1** was established. In addition, the angeloyl could be positioned at C-3 in compound **1**, owing to the HMBC correlation of H-3 (*δ*_H_ 4.76) to C-1′ (*δ*_C_ 167.7). The relative configuration of **1** was characterized by interpretation of the ROESY spectrum (Fig. 2). The observation of ROESY correlations of H-3 with H-5 (*δ*_H_ 1.72, dd, *J* = 12.4, 4.6 Hz), and Me-14 to Me-15, indicated that H-3 and H-5 were in the same orientation, and Me-14 and Me-15 were in the opposite direction against H-3 and H-5. Based on biosynthetic grounds, the absolute configuration of **1** was suggested as dodelates A–E [3] and coexisting compounds **2**–**5**. Therefore, compound **1** was finally assigned as shown in Fig. 1, and named dobinin O. A hypothetical biosynthetic pathway for compound **1** was proposed as shown in Scheme 1.

By comparison of their spectroscopic data and physicochemical properties with those reported in the literature, the four known eudesmane sesquiterpenoids were identified as dobinin A (**2**) [4], 3β-angeloyloxy-4α,8β-dihydroxy-eudesm-7(11)-en-8α,12-olide (**3**) [5], dobinin C (**4**) [4], and furanoeudesmane B (**5**) [6], respectively (Fig. 2).

Compound **1** was evaluated for its in vivo antimalarial activity against *Plasmodium yoelii* BY265RFP in mice, according to a four-day suppressive test [3]. As shown in Table 2, compound **1** exhibited moderate antimalarial activity with the inhibition ratio of 17.8 ± 13.3% at the dose of 30 mg/kg/day. This was further confirmed by the features of relief of hepatomegaly, increase of number of erythrocyte and content of hemoglobin, and recovery of abdominal temperature when the infected mice were treated with compound **1** (Tables S1–S3, Supporting Information). Furthermore, the immunomodulatory effect of compound **1** on the host response was also evaluated by the levels of splenic CD4^+^CD25^+^ regulatory T cells (Tregs), IL-10, IL-12, IFN-*γ* and IgG. As shown in Tables S4 and S5 (in Electronic supplementary material), IL-12, IFN-*γ* and immunosuppressive CD4^+^CD25^+^ Tregs [7, 8] from spleen significantly increased or decreased upon **1** administration at 30 mg/kg/day like those of administration of chloroquine diphosphate, suggesting that **1** could induce apoptosis of parasitized erythrocytes by elevating the level of IL-12 [9, 10], and the immunity of the infected mice could be recovered by **1** treatment.

**Table 2:** In vivo antimalarial activity of compound **1** against *P. yoelii* BY265RFP ($\overline{x} \pm s$, *n* = 6)

Compound	Dose (mg/kg/day)	Inhibition (%)
CQ	10	98.4 ± 0.6
**1**	30	17.8 ± 13.3

*CQ* chloroquine diphosphate

## Experimental Section

### General

NMR spectra (1D and 2D NMR) were recorded on a Bruker Avance III-400 instrument (Bruker, Faellanden, Switzerland) with TMS as an internal reference. HRESIMS data were obtained on a Dionex Ultimate 3000 LC System (Thermo Fisher Scientific, Sunnyvale, USA) coupled in series to a Bruker Compact quadrupole time-of-flight (QTOF) mass spectrometer (Bruker, Bremen, Germany). Optical rotations were determined on a SGW-3 automatic polarimeter (Shanghai INESA Physico optiacal instrument Co., Ltd, Shanghai, P. R. China). UV data were obtained on a TU-1901 UV/Vis spectrophotometer (Beijing Purkinje General Instrument Co. Ltd., Beijing, P. R. China). IR spectra were recorded by a Nicolet 380 FT-IR spectrophotometer (Thermo Scientific, Madison, WI, USA) with KBr pellets. Silica gel (Qingdao Marine Chemical Ltd., Qingdao, P. R. China) and Sephadex LH-20 (Amersham Biosciences, Uppsala, Sweden) were used for open column chromatography.

### Plant Material

Dried and peeled roots of *Dobinea delavayi* (Baill.) Baill. were purchased from the herbal medicine market of Eryuan county, Dali, Yunnan Province, P. R. China in October 2017. The material was identified by Dr. Bei Jiang, a professor from Dali University, P. R. China. A voucher specimen (No. 20171008-1) has been deposited at the Institute of Materia Medica, Dali University.

### Extraction and Isolation

The roots of *D. delavayi* (1.1 kg) was extracted with 80% ethanol at room temperature (5 × 10 L, each for 24 h), and the extract solutions were combined and concentrated under reduced pressure. Subsequently, the resulting residue (180 g) was suspended in water and partitioned with ethyl acetate. The EtOAc soluble portion (39 g) was fractionated by a silica gel column chromatography (CC) eluting with a gradient solvent system of petroleum ether (PE)/acetone (50:1 to 0:1) to give seven major fractions (Fr. A–Fr. G). Fr. A (15 g) was subsequently separated on a silica gel CC (PE/acetone, 50:1) to give twelve subfractions Fr. A-1 to Fr. A-12. Fr. A-3 emerged as some colorless crystals after several hours settling at room temperature, then repeatedly washed with methanol to yield compound **5** (20 mg). The residual solution of Fr. A-3 was subjected to repeated Sephadex LH-20 CC (acetone) to afford compound **1** (52 mg). Fr. C (3.4 g) was separated on a silica gel CC with PE/acetone (10:1), and then was further purified by a Sephadex LH-20 CC (acetone) to give compound **2** (25 mg). Fr. D (3.2 g) emerged as some colorless crystals after several hours settling at room temperature, then repeatedly washed with methanol to yield compound **3** (28 mg). The residual solution of Fr. D was separated by CC on silica gel (PE/EtOAc, 30:1) and Sephadex LH-20 (acetone) successively, to give compound **4** (8 mg).

Dobinin O (**1**)*:* white powder; $\alpha_{{\text{D}}}^{25}$ − 41.4 (*c* 0.10, MeOH); UV (MeOH) *λ*_max_ (log *ε*) 208.0 (3.72), 288.0 (3.40) nm; IR (KBr) *ν*_max_ 3463, 2935, 1716, 1612, 1366, 1247, 1160, 1080, 671 cm^−1^; ^1^H and ^13^C NMR data, see Table 1; HRESIMS *m/z* 335.1866 [M‒H]^−^ (calcd for C_19_H_27_O_5_, 335.1864).

### Antimalarial Activity

The antimalarial assay was performed according to a four-day suppressive test as we previously described [3].

## Electronic supplementary material

Below is the link to the electronic supplementary material.

Supplementary file1 (DOCX 1886 kb)
