# Supplementary material for: A New Antimalarial Noreudesmane Sesquiterpenoid from Dobinea delavayi
Source: Nat Prod Bioprospect. 2020 Feb 24;10(2):101–4. doi: 10.1007/s13659-020-00234-4 (PMC7176782; doi:10.1007/s13659-020-00234-4)
Supplement: Supplementary file 1 — Supplementary file1 (DOCX 1886 kb) [file 13659_2020_234_MOESM1_ESM.docx]

**Supporting Information**

**A New Antimalarial Noreudesmane Sesquiterpenoid from** ***Dobinea delavayi***

Xiu-Rong Wu^1,2^ ⋅ Yi Shen^1,2^ ⋅ Shu-Jun Cui^1,2^ ⋅ Xiao-Lei Luo^1,2^ ⋅ Chao-Jiang Xiao^2^ ⋅ Bei Jiang^1^

^1^ Institute of Materia Medica, Dali University, Dali 671000, People’s Republic of China

^2^ College of Pharmacy and Chemistry, Dali University, Dali 671000, People’s Republic of China

Xiu-Rong Wu and Yi Shen contributed equally to this work.

**Corresponding author:**

Prof. Dr. Bei Jiang, Institute of Materia Medica, Dali University, Xueren Road 2, Dali 671000, China. E-mail: jiangbei@dali.edu.cn, Phone: +86 872 225 7316; Fax: +86 872 225 7401

Dr. Chao-Jiang Xiao, College of Pharmacy and Chemistry, Dali University, Xueren Road 2, Dali 671000, China. E-mail: xiaochaojiang@yeah.net, Phone: +86 872 225 7259; Fax: +86 872 225 7401

**CONTENTS**

[**Figure S1.** ^1^H NMR spectrum for compound **1** (400 MHz, CD_3_COCD_3_) 1](#_Toc28504909)

[**Figure S2.** ^13^C NMR and DEPT spectra for compound **1** (100 MHz, CD_3_COCD_3_) 2](#_Toc28504910)

[**Figure S3.** HSQC spectrum for compound **1** (*f*_2_ 400 MHz, *f*_1_ 100 MHz, CD_3_COCD_3_) 3](#_Toc28504911)

[**Figure S4.** HMBC spectrum for compound **1** (*f*_2_ 400 MHz, *f*_1_ 100 MHz, CD_3_COCD_3_) 4](#_Toc28504912)

[**Figure S5.** Expansion of HMBC spectrum for compound **1** (*f*_2_ 400 MHz, *f*_1_ 100 MHz, CD_3_COCD_3_) 5](#_Toc28504913)

[**Figure S6.** ^1^H-^1^H COSY spectrum for compound **1** (400 MHz, CD_3_COCD_3_) 6](#_Toc28504914)

[**Figure S7.** ROESY spectrum for compound **1** (400 MHz, CD_3_COCD_3_) 7](#_Toc28504915)

[**Figure S8.** IR spectrum of compound **1** 8](#_Toc28504916)

[**Figure S9.** UV spectrum of compound **1**. 9](#_Toc28504917)

[**Figure S10.** HR-ESI-MS spectrum for compound **1** 10](#_Toc28504918)

[**Table S1.** Effect of compound **1** on organ coefficients of *P. yoelii* BY265RFP infected mice 11](#_Toc28504919)

[**Table S2.** Effect of compound **1** on hematologic parameters of *P. yoelii* BY265RFP infected mice 11](#_Toc28504920)

[**Table S3.** Effect of compound **1** on abdominal temperatures of *P. yoelii* BY265RFP infected mice 11](#_Toc28504921)

[**Table S4.** Effect of compound **1** on IL-10, IL-12, IFN-γ and IgG production of *P. yoelii* BY265RFP infected mice 12](#_Toc28504922)

[**Table S5.** Effect of compound **1** on the proportion of splenic CD4^+^CD25^+^ Tregs within all CD4^+^ Cells from *P. yoelii* BY265RFP infected mice 12](#_Toc28504923)

**Figure S1.** ^1^H NMR spectrum for compound **1** (400 MHz, CD_3_COCD_3_).

**Figure S2.** ^13^C NMR and DEPT spectra for compound **1** (100 MHz, CD_3_COCD_3_).

**Figure S3.** HSQC spectrum for compound **1** (*f*_2_ 400 MHz, *f*_1_ 100 MHz, CD_3_COCD_3_).

**Figure S4.** HMBC spectrum for compound **1** (*f*_2_ 400 MHz, *f*_1_ 100 MHz, CD_3_COCD_3_).

**Figure S5.** Expansion of HMBC spectrum for compound **1** (*f*_2_ 400 MHz, *f*_1_ 100 MHz, CD_3_COCD_3_).

**Figure S6.** ^1^H-^1^H COSY spectrum for compound **1** (400 MHz, CD_3_COCD_3_).

**Figure S7.** ROESY spectrum for compound **1** (400 MHz, CD_3_COCD_3_).

**Figure S8.** IR spectrum of compound **1**.


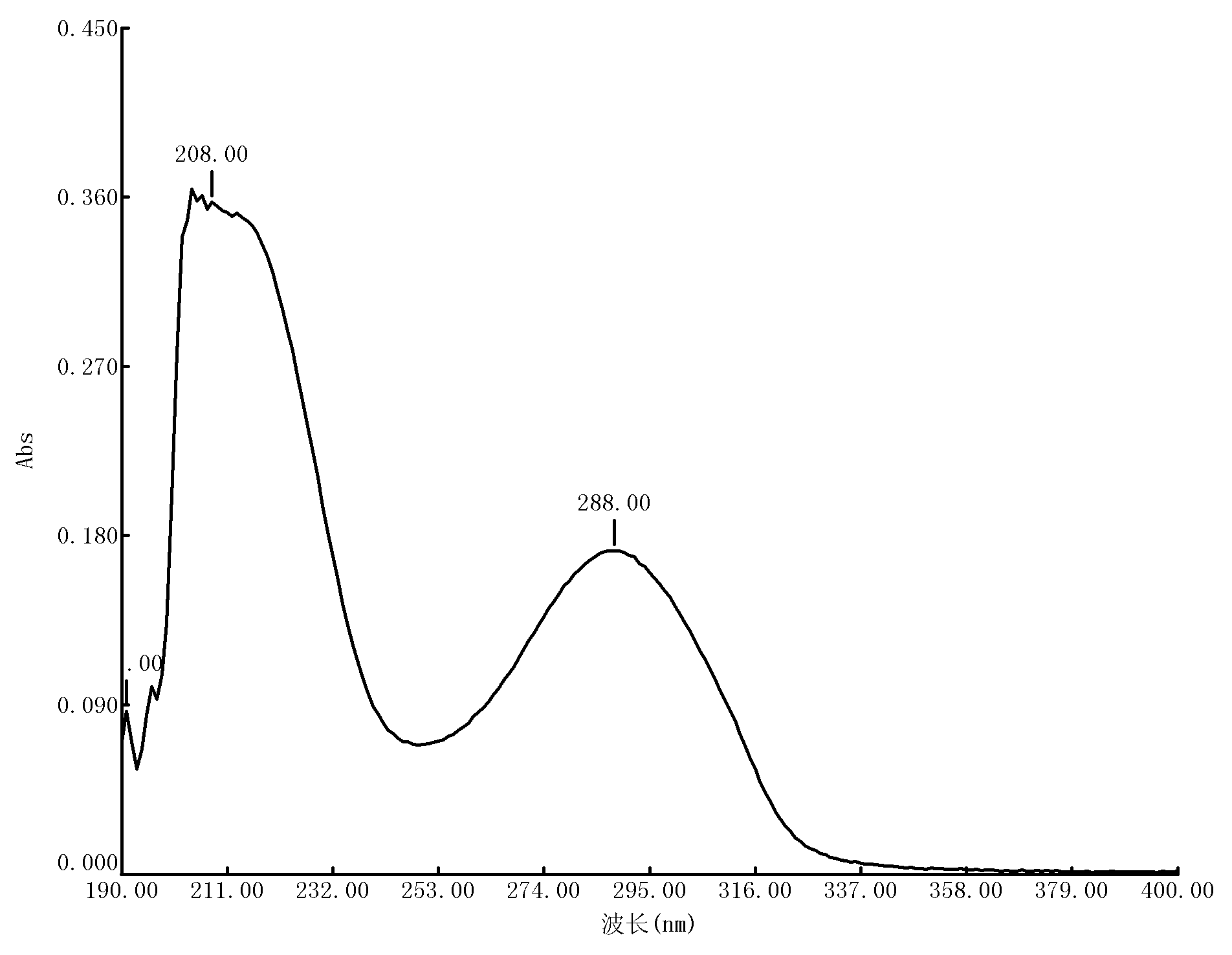


**Figure S9.** UV spectrum of compound **1**.

**Figure S10.** HR-ESI-MS spectrum for compound **1**.

**Table S1.** Effect of compound **1** on organ coefficients of *P. yoelii* BY265RFP infected mice ($\bar{\text{x}}$ ± *s*, *n* = 6).

| compound | dose (mg/kg/day) | heart | liver | lung | kidney | brain | testis |
| --- | --- | --- | --- | --- | --- | --- | --- |
| normal | - | 0.63 ± 0.07 | 5.79 ± 0.10 | 0.82 ± 0.03 | 1.62 ± 0.09 | 1.23 ± 0.06 | 0.70 ± 0.02 |
| control | - | 0.60 ± 0.05 | 7.30 ± 0.32 | 1.01 ± 0.22 | 1.53 ± 0.08 | 1.44 ± 0.23 | 0.63 ± 0.06 |
| CQ | 10 | 0.59 ± 0.03 | 6.10 ± 0.53* | 0.86 ± 0.20* | 1.41 ± 0.06* | 1.63 ± 0.33* | 0.61 ± 0.09 |
| **1** | 30 | 0.65 ± 0.09 | 6.31 ± 0.46* | 0.82 ± 0.12* | 1.43 ± 0.11* | 1.25 ± 0.10* | 0.61 ± 0.07 |

The organ coefficients were calculated as follows: organ coefficient = 100 × mass of organ / weight of mouse. Normal: uninfected mice treated with 0.5% CMC-Na; control: infected mice treated with 0.5% CMC-Na; CQ: chloroquine diphosphate. Compared with the control group, **P* < 0.05.

**Table S2.** Effect of compound **1** on hematologic parameters of *P. yoelii* BY265RFP infected mice ($\bar{\text{x}}$ ± *s*, *n* = 6).

| compound | dose (mg/kg/day) | leukocyte (10^9^/L) | lymphocyte (10^9^/L) | erythrocyte (10^12^/L) | hemoglobin (g/L) |
| --- | --- | --- | --- | --- | --- |
| normal | - | 3.41 ± 0.20 | 2.36 ± 0.20 | 2.35 ± 0.25 | 249.00 ± 38.00 |
| control | - | 5.36 ± 0.27 | 4.39 ± 0.30 | 0.75 ± 0.24 | 40.00 ± 7.00 |
| CQ | 10 | 3.01 ± 0.19* | 2.61 ± 0.25* | 2.25 ± 0.15* | 234.00 ± 13.00* |
| **1** | 30 | 5.20 ± 0.20 | 3.92 ± 0.32* | 1.16 ± 0.18* | 128.00 ± 22.00* |

The hematologic parameters were analysed by a XFA6130 automatic blood cell analyzer (Nanjing Pulang Biologic Technology Co., Ltd., Nanjing, P. R. China). Normal: uninfected mice treated with 0.5% CMC-Na; control: infected mice treated with 0.5% CMC-Na; CQ: chloroquine diphosphate. Compared with the control group, **P* < 0.05.

**Table S3.** Effect of compound **1** on abdominal temperatures of *P. yoelii* BY265RFP infected mice ($\bar{\text{x}}$ ± *s*, *n* = 6).

| compound | dose (mg/kg/day) | D1 (^o^C) | D4 (^o^C) |
| --- | --- | --- | --- |
| normal | - | 36.4 ± 0.2 | 36.5 ± 0.2 |
| control | - | 35.8 ± 0.4 | 32.6 ± 0.8 |
| CQ | 10 | 35.6 ± 0.6 | 36.5 ± 0.2* |
| **1** | 30 | 35.5 ± 0.3 | 34.3 ± 0.2* |

Abdominal temperatures of all mice were recorded by a portable infrared thermometer (CK-T1503, Shenzhen ChangKun Technology Co., Ltd., Shenzhen, P. R. China). Normal: uninfected mice treated with 0.5% CMC-Na; control: infected mice treated with 0.5% CMC-Na; CQ: chloroquine diphosphate; D1: the temperature was measured on day 2 after infection; D4: the temperature was measured on day 5 after infection. Compared with the control group, **P* < 0.05.

**Table S4.** Effect of compound **1** on IL-10, IL-12, IFN-γ and IgG production of *P. yoelii* BY265RFP infected mice ($\bar{\text{x}}$ ± *s*, *n* = 3).

| compound | dose (mg/kg/day) | IL-10 (ng/L) | IL-12 (ng/L) | IFN-γ (ng/L) | IgG(mg/mL) |
| --- | --- | --- | --- | --- | --- |
| normal | - | 235.07 ± 25.00 | 154.26 ± 23.10 | 366.33 ± 20.16 | 8.29 ± 1.73 |
| control | - | 196.05 ± 16.00 | 188.07 ± 28.00 | 414.00 ± 24.00 | 54.30 ± 12.88 |
| CQ | 10 | 178.57 ± 28.50 | 566.33 ± 36.01* | 163.33 ± 23.50* | 396.54 ± 46.50* |
| **1** | 30 | 240.40 ± 28.36* | 237.03 ± 27.00* | 259.00 ± 29.00* | 0.80 ± 0.10* |

Cytokines IL-10, IL-12 and IFN-*γ*, and antibody IgG in serum relating to immune regulation were tested by solid phase sandwich enzyme-linked immunosorbent assay (ELISA) according to the manufacturer’s protocol. Normal: uninfected mice treated with 0.5% CMC-Na; control: infected mice treated with 0.5% CMC-Na; CQ: chloroquine diphosphate. Compared with the control group, **P* < 0.05.

**Table S5.** Effect of compound **1** on the proportion of splenic CD4^+^CD25^+^ Tregs within all CD4^+^ Cells from *P. yoelii* BY265RFP infected mice ($\bar{\text{x}}$ ± *s*, *n* = 3).

| compound | dose (mg/kg/day) | percentage (%) |
| --- | --- | --- |
| normal | - | 6.0 ± 0.4 |
| control | - | 11.2 ± 0.5 |
| CQ | 10 | 6.7 ± 0.6* |
| **1** | 30 | 7.0 ± 0.9* |

Splenic CD4^+^CD25^+^ Tregs were detected as a reported method (Farsam V., et al. *Int. Immunopharmacol.* **2011**, *11*, 1802-1808.) by flow cytometry on a FACSCalibur flow cytometer (Becton Dickinson, San Jose, CA, USA). Normal: uninfected mice treated with 0.5% CMC-Na; control: infected mice treated with 0.5% CMC-Na; CQ: chloroquine diphosphate. Compared with the control group, **P* < 0.05.
